# Bringing the Line Home: A Perspective on Establishing At-Home PICC (Peripherally Inserted Central Catheter) Insertion in Oncology Patients

**DOI:** 10.3390/clinpract16080138

**Published:** 2026-07-27

**Authors:** Orestis Ioannidis, Antonia Aikaterini Bourtzinakou, Elissavet Anestiadou, Evangelia Ioannidou, Konstantinos Siozos, Georgios Gemousakakis, Stefanos Bitsianis, Savvas Symeonidis, Efstathios Kotidis, Manousos Georgios Pramateftakis, Ioannis Mantzoros, Stamatios Angelopoulos

**Affiliations:** 4th Department of Surgery, School of Medicine, Faculty of Health Sciences, Aristotle University of Thessaloniki, G. Papanikolaou Hospital, 57010 Thessaloniki, Greece; katerbourtzi@gmail.com (A.A.B.); elissavetxatz@gmail.com (E.A.); liaioannidou78@gmail.com (E.I.); kwstassiwzos@hotmail.gr (K.S.); gtgeorge@rocketmail.com (G.G.); sbitsiani@gmail.com (S.B.); simeonidissavvas@yahoo.com (S.S.); mpramateftakis@hotmail.com (M.G.P.); imanvol@gmail.com (I.M.); saggelopoulos@auth.gr (S.A.)

**Keywords:** vascular access, oncology patients, peripherally inserted central catheters (PICC), at-home PICC

## Abstract

**Background and Objectives:** Vascular access in cancer patients with limited mobility remains a persistent clinical challenge, although both devices and insertion methods have improved considerably. We present our clinical experience with at-home PICC placement, supported by real-world data from routine practice, in oncology patients unable to attend hospital care, exploring its feasibility, safety, and practical applicability. **Materials and Methods:** All PICC insertions were performed at patients’ bedside by a trained surgeon using sterile technique. A single- or double-lumen PICC catheter was inserted under ultrasound guidance, magnetic tracking, and intracavitary ECG confirmation to secure optimal venous entry and accurate tip positioning. **Results:** A total of 28 PICC lines were successfully inserted in 26 oncologic patients. Two patients required catheter re-insertion, one following accidental removal and one due to infection. In 26 of 28 cases (92.9%), insertions were completed on the first attempt, while two cases required a second attempt. Insertion in the Green Zone per the ZIM classification was achieved in 26 of 28 cases (92.9%), and only two required insertions in the Yellow Zone. The basilic vein was chosen in 75% of patients. A total of 23 single-lumen and five double-lumen catheters were inserted. In terms of complications, four cases of systemic infection occurred more than 15 days after insertion and were not considered insertion-related; two patients developed localized edema requiring anticoagulation, and four catheter occlusions were managed conservatively. **Conclusions:** At-home PICC placement appears to be a feasible and clinically applicable approach when performed by qualified and credentialed professionals. These observations, derived from real-world clinical experience, suggest a potential role for this model in selected patient populations. Using proper sterile technique and real-time tip confirmation tools, this intervention may contribute to improved continuity of care and reduced hospital visits in selected patients. These observations suggest a potential role for expanding outpatient vascular access programs in selected patient populations.

## 1. Introduction

The global prevalence of cancer continues to rise, with approximately 20 million new cases diagnosed worldwide in 2022, a number projected to reach nearly 35 million annually by 2050 [1]. A significant number of oncology patients consist of older and frail individuals, with reduced functional capacity and mobility limitations, who require prolonged supportive treatment outside the hospital setting. In this context, the ability to establish vascular access devices directly at home may represent a robust solution that will expand the scope of vascular care for this growing population.

The use of peripherally inserted central catheters (PICC) for the administration of fluids, medications, supplements, and parenteral nutrition has been well-documented in numerous scientific studies in recent years [2]. This mode of vascular access is particularly important for patients with malignancies or other chronic conditions, as PICC lines significantly enhance their daily care and overall quality of life [3]. In contemporary vascular access practice, the procedural approach to PICC line placement in even the most challenging cases, such as cancer patients, refers to the line being placed in an in-hospital setting. The primary objective is to accommodate diverse and complex clinical scenarios while ensuring a safe and smooth placement of the catheter.

Adult cancer patients almost invariably develop nutritional deficiencies or feeding impairments during the course of their disease. As per the guidelines for “general concepts of treatment relevant to all cancer patients” of the European Society for Clinical Nutrition and Metabolism—ESPEN, patients with advanced cancer should implement nutritional interventions after prognosis has been evaluated. In this regard, patients with an estimated life expectancy of several months or more should receive appropriate nutritional supplementation [4].

Considering these principles, we described the current situation in Greece and identified several challenges. The country presents a unique challenge for centralized healthcare systems due to its demographic and geographic profile. According to the most recent data (2021), our country exhibits one of the oldest populations in Europe, with approximately 22.9% of citizens aged 65 years or older [5]. Furthermore, despite high urbanization rates, a significant portion of people, approximately 21%, reside in rural and remote areas, including islands and mountainous regions [6]. The following issue consistently emerges: How can patients of an aging, often frail population, with great difficulty accessing larger medical institutions, obtain adequate venous access when needed?

The gradual and consistent advancement of PICC insertion techniques, along with improvements in the materials used, including ultrasound guidance and improved catheter materials, has enabled the placement of these catheters in a broader range of patients. This includes individuals who were previously classified as having “difficult venous access” and limited vascular access options [7]. Assessing the feasibility of such interventions in patients with limited mobility, bedridden individuals, and elderly patients with malignancies who often encounter significant difficulties with hospital-based interventions remains an essential component of optimizing their care.

Despite the technological and procedural advancements, no official state-run service exists for the placement of peripherally inserted central lines in these patients in Greece. Although certain publicly organized home-nursing programs or medication-delivery services exist, they remain limited in scope and capacity [8]. Consequently, the responsibility for establishing venous access typically falls to private clinicians or small regional hospitals, which often have limited experience in performing such procedures due to their lower patient volumes. This organizational gap is particularly consequential for frail oncology patients who would benefit from reliable venous access outside tertiary centers.

At our institution, the vascular access team has placed more than 3500 PICCs in hospitalized patients over the past 10 years, including 198 used specifically for home parenteral nutrition, while approximately 500 were bedside insertions. Building on this experience with a broad range of central venous access devices, we undertook a novel initiative: the at-home insertion of PICCs. This service, led by a dedicated surgeon with extensive vascular access training, was applied in a small cohort of 26 patients across Northern Greece. Although outpatient vascular access and home-based catheter care are well established, published data specifically addressing PICC insertion performed entirely in the home setting remain extremely limited. To date, only a small number of reports, including the pilot study by Keller et al. [9], have explored this approach, highlighting the rarity of its implementation. An additional distinguishing feature of this work is the care delivery model. Unlike most published studies, which describe team-based or institutionally supported vascular access services, the procedures in this series were performed by a single, experienced operator. This highlights a pragmatic approach that may be particularly relevant in healthcare systems lacking dedicated vascular access teams.

The aim of this article is to present our perspective on at-home PICC placement in adult oncology patients, based on direct clinical experience supported by structured real-world data. This work is not intended as a formal observational or comparative study with hospital-based PICC placement, but rather as a feasibility pilot experience presented within a practice-based perspective, aiming to highlight an underexplored clinical approach, describe the procedural feasibility and safety of at-home catheter insertion, demonstrate that it is technically achievable and applicable in a selected patient population, and share practical insights for its implementation. Through our pilot experience, we aim to highlight the need for a shift in national healthcare policy and reimbursement structures to enable such services to be performed by appropriately trained and experienced professionals and to become widely accessible to all patients in need.

This manuscript should not be interpreted as a prospective study, but as a perspective grounded in clinical experience and supported by structured observational retrospectively reviewed data collected during routine clinical practice.

## 2. Feasibility Pilot Experience

This work is presented as a perspective supported by structured real-world data derived from routine clinical practice. The clinical data were recorded prospectively during the performance of at-home PICC insertions and were subsequently analyzed descriptively to support the authors’ viewpoint. This approach does not represent a predefined, hypothesis-driven observational study, but rather a practice-based analysis intended to describe feasibility, safety, and implementation aspects of at-home PICC placement. No comparative analysis with hospital-based or outpatient insertion models was intended. Where applicable, elements of the principles outlined in the STROBE statement for observational studies were followed to ensure transparency and clarity [10]. All procedures were conducted in accordance with the Declaration of Helsinki and approved by the appropriate institutional ethics committee [11]. Written informed consent was obtained from all patients prior to the procedure as described in the pre-procedural assessment. The data presented are descriptive and reflect real-world clinical practice, intended to support a clinical perspective rather than to serve as a formal comparative analysis, and to assess whether at-home PICC placement is feasible, effective, and safe for this specific patient population.

A total of 26 patients underwent at-home PICC insertion between 2019 and 2025. The available data for each patient were derived from the routine follow-up performed as standard care, rather than from a protocol designed specifically for research purposes. All procedures were performed by an attending general surgeon with extensive vascular access experience, who traveled to each patient’s home using a privately owned vehicle with all necessary equipment and materials, in order to perform the PICC placement in the home setting, either independently or with the assistance of an experienced nurse. In five cases, a second general surgeon, with some vascular access experience, observed the procedure for teaching purposes, while in 13 cases, the specialized nurse provided by the supplying company was also present during the insertion. In all cases, the patient’s caregivers’ help proved to be vital in the successful outcome of this endeavor, ensuring a smooth and safe workflow in the home environment.

### 2.1. Pre-Procedural Assessment and Preparation

Prior to catheter placement, an in-depth discussion was conducted with both the patients and their caregivers, whenever feasible. A thorough medical history was obtained, and informed consent was duly acquired. This research was conducted in accordance with the ethical principles of the World Medical Association Declaration of Helsinki. To guarantee that the shift to a home setting did not compromise patient safety, our procedural workflow closely mirrored the SIP (Safe Insertion of PICCs) protocol, incorporating all key pillars, such as pre-procedural ultrasound assessment, maximal barrier precautions, and intra-procedural ECG-based tip location [12]. This standardized approach allowed us to maintain hospital-equivalent safety standards at the patient’s bedside.

**Figure 1:**
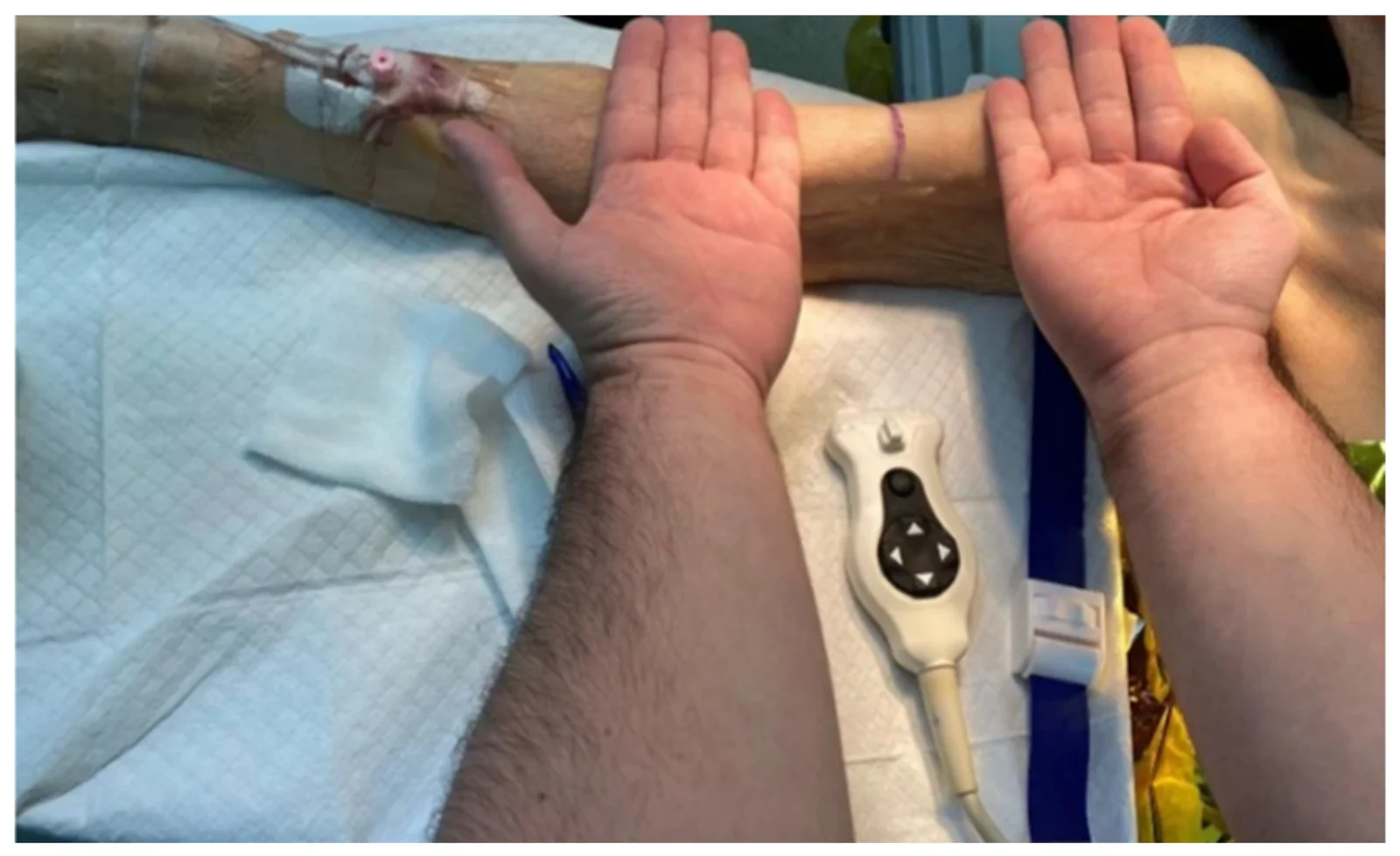
Zone Insertion Method (ZIM).

The inclusion of technical images is intended to support the ‘tips and tricks’ nature of this perspective, facilitating practical understanding and reproducibility of the procedure in the home setting. The procedure was performed at the patient’s residence, with the patient lying in supine position on a bed, which was ideally double, to provide adequate working space. The surgeon was seated on the side of the selected upper limb for catheter insertion; this was most commonly the right arm, while the ultrasound device was positioned on the opposite side, facing the operator, typically placed on a bedside table or any stable surface available. In 24 of 26 cases (92.3%), PICCs were inserted in the Green Zone according to the Zone Insertion Method (ZIM™) (Figure 1) [13]. This approach divides the upper arm into three distinct zones marked by colors—Green, Yellow, and Red—to guide safe venous access. The Green Zone, located in the mid-upper arm, is considered ideal for insertion due to optimal vein diameter and minimal risk of injury to nearby nerves or arteries. In the few cases requiring placement within the Yellow Zone, a subcutaneous tunnel was intentionally not created. This decision was driven by two factors. First, in all such cases, the exit site of the catheter was located in the distal third of the Yellow Zone, which was considered acceptable. Second, the management of potential tunneling-related complications (hemorrhage/hematoma, neurovascular injury, catheter kinking) could not be easily or adequately addressed in the home setting, in terms of safety and efficacy.

**Figure 2:**
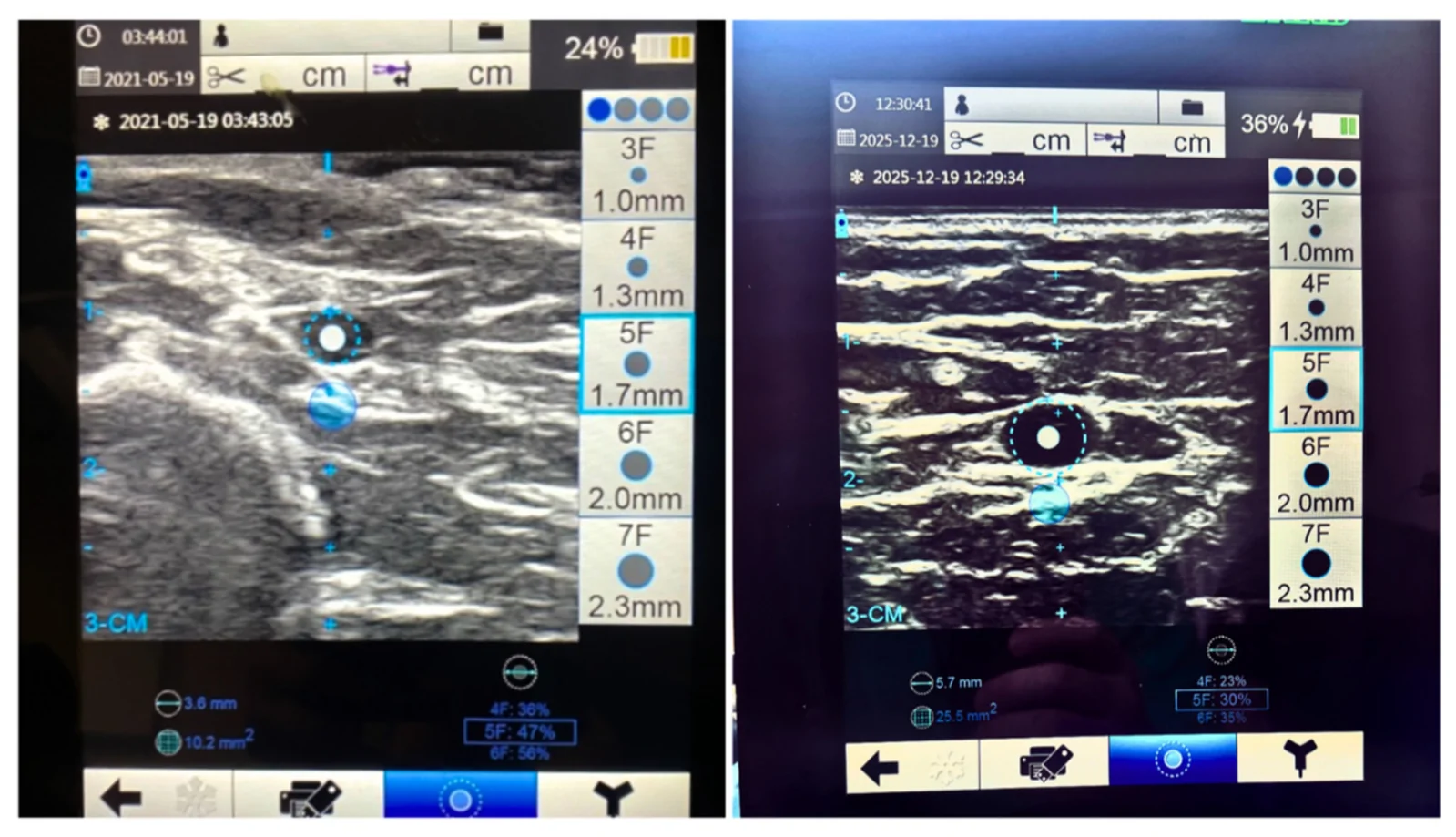
**Comparison of cross-sectional area of vessel to the catheter using U/S guidance.** Real-time calculation of vessel occupancy. In this example, the 47% occupancy is considered suboptimal/contraindicated, as a ratio of ≤33% is required for safe catheter placement.

**Figure 3:**
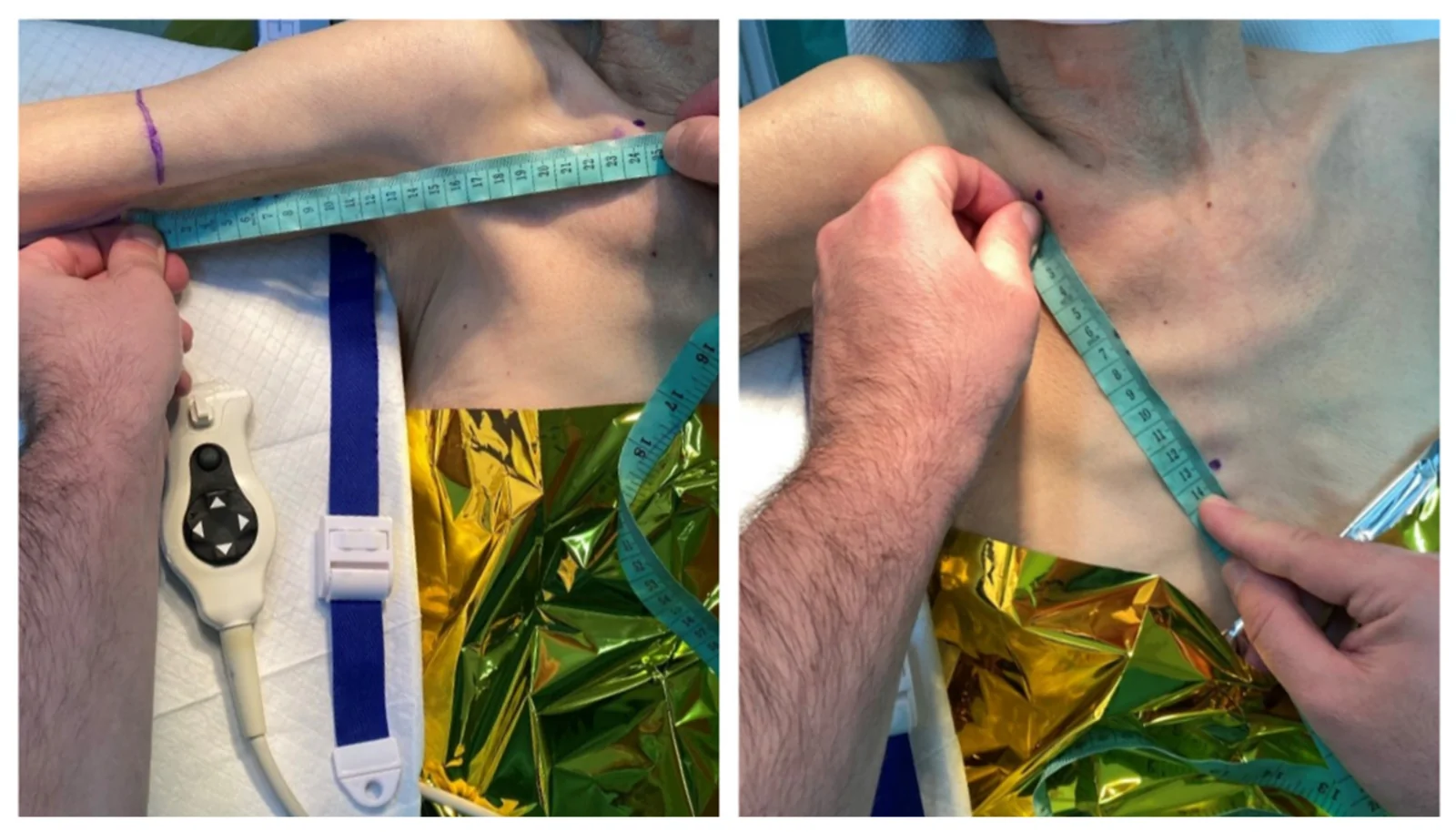
Pre-procedural estimation of catheter length. The operator measures from the insertion site to the mid-clavicle and vertically to the third intercostal space.

The basilic or brachial vein was selected and pre-marked after thorough ultrasound evaluation of both arms, ensuring that the vessel diameter was at least three times that of the catheter of choice. The ultrasound device also allowed for a direct comparison between the cross-sectional area of the selected vessel and that of the catheter, expressed as a percentage (Figure 2). It is also important to note that the surgeon carried a supply of materials, including a second and third PICC line, along with all necessary consumables, including a microintroducer set, in order to ensure uninterrupted workflow. Pre-insertion measurement was also performed to ensure accurate tip placement. Using a measuring tape, the distance was mapped from the puncture site to the right clavicular mid-point and down to the third intercostal space (Figure 3).

To prevent gross contamination prior to the procedure, the surgeon wore a surgical cap, mask, and shoe covers. Thorough hand washing was performed in the domestic bathroom, followed by further disinfection using an alcohol-based antiseptic (Sterillium^®^ BODE Chemie GmbH, Hamburg, Germany). After donning a sterile gown, a double gloving technique was employed: the outer pair was used during skin antisepsis and subsequently removed, leaving the inner sterile pair for the catheter insertion. To establish a sterile field and maintain an aseptic environment, a full-tray sterile drape was utilized. PICCs used were 5Fr single-lumen PowerGroshong™ PICC Catheter (Bard Access Systems, Inc., Salt Lake City, UT, USA) (silicone construction, distally valved with a three-way valve) or a 5F double-lumen open-ended PowerPICC™ Catheter (Bard Access Systems, Inc., Salt Lake City, UT, USA), along with a Sherlock 3CG+™ TCS system (Becton, Dickinson and Company, Franklin Lakes, NJ, USA)., designed to aid in tip positioning of catheters. The number of lumens was determined based on the type and volume of intravenous therapy the patient was expected to receive.

### 2.2. Catheter Insertion Technique

The procedural steps were standardized and consistently followed by the surgeon in every case. These included field sterilization using full draping and skin disinfection with a solution of 70% *v*/*v* isopropyl alcohol and 2% *w*/*v* chlorhexidine gluconate. Patients were positioned supine at the edge of the bed opposite the insertion site, with the target arm abducted and externally rotated, being fully supported by the rest of the bed.

**Figure 4:**
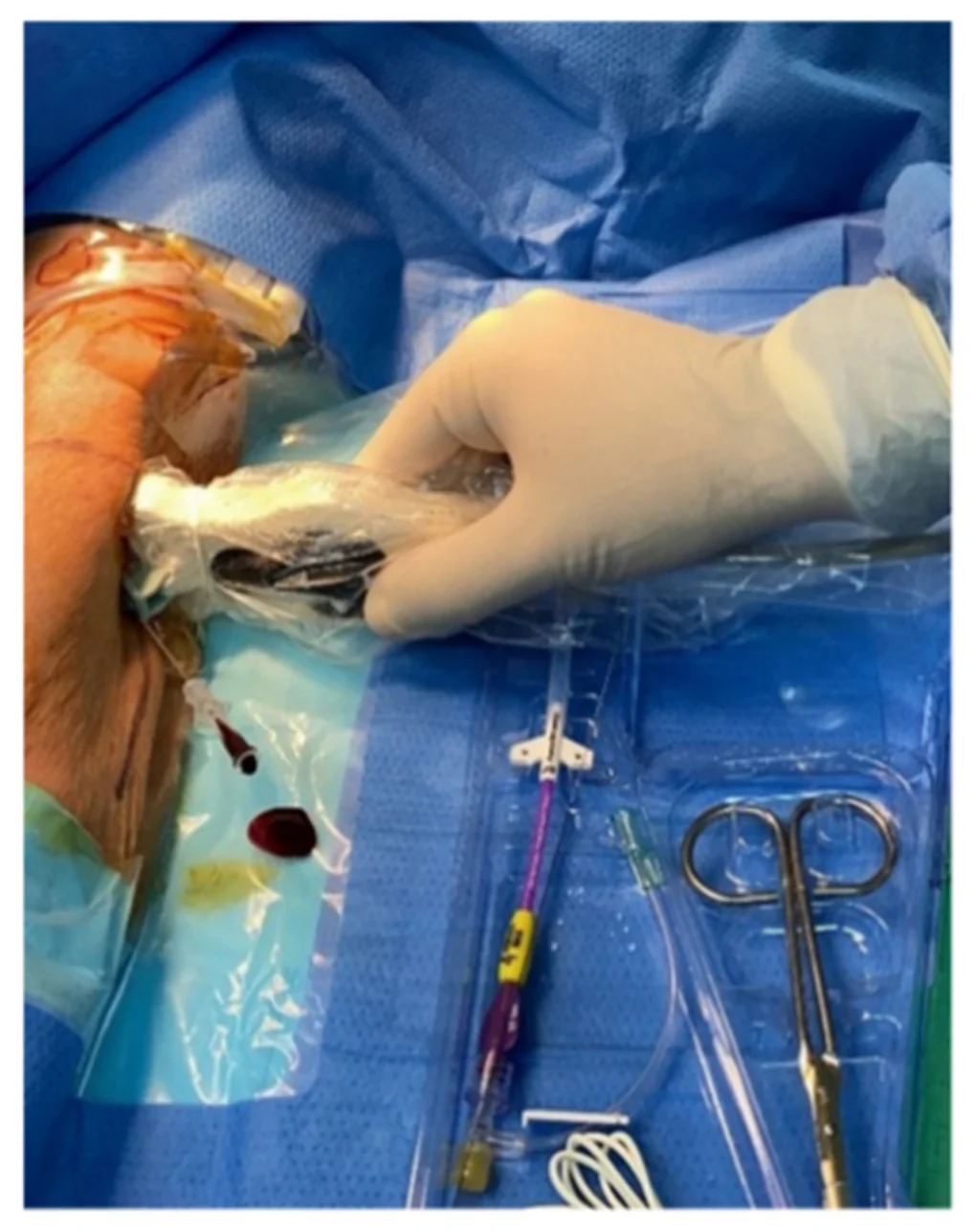
Confirmation of blood return with the tourniquet tightened.

Catheterization was performed at the point where the vessel demonstrated its maximum size, preferably on the right arm, after vascular assessment was performed using the Site-Rite^®^ 8 Ultrasound System (Bard Access Systems, Inc., Salt Lake City, UT, USA). To facilitate accurate venipuncture, the ultrasound transducer is equipped with a needle guide, which significantly simplifies needle alignment and advancement into the target vessel. Upon needle insertion, it is imperative to observe a clear blood return. To facilitate this, the tourniquet remains secured throughout the venipuncture process, allowing for immediate visual confirmation of successful vessel access (Figure 4).

**Figure 5:**
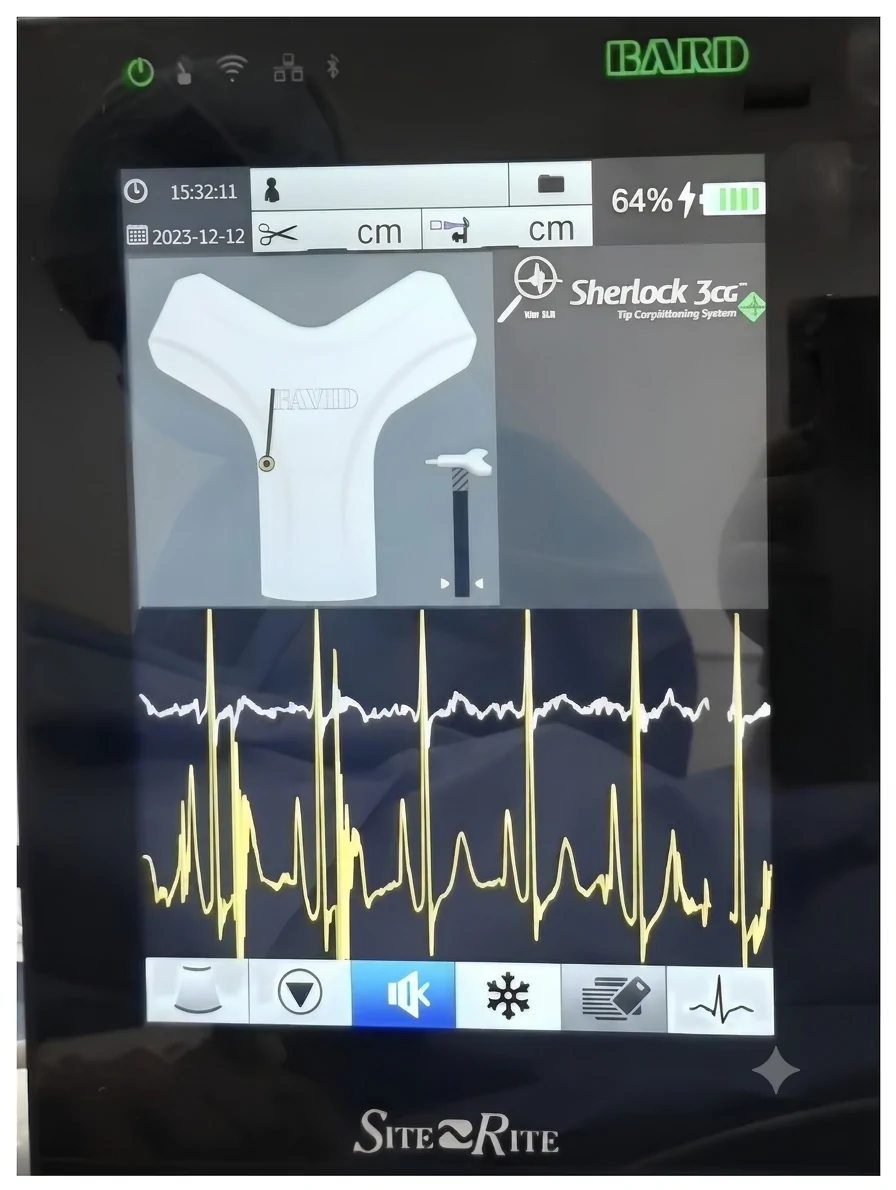
**Real-time catheter navigation information with magnet navigation and/or ECG-based navigation.** Prominent P-wave amplitude was observed, consistent with the increasing atrial signal as the catheter tip approached the cavo-atrial junction. The stable magnetic signal confirmed intravascular orientation and central venous trajectory.

To confirm the catheter tip position, a magnetic tracking system combined with ECG-based peripheral central catheter tip verification technology (Sherlock 3CG+™ TCS, Bard Access Systems, Inc., Salt Lake City, UT, USA) was employed (Figure 5). Catheter stabilization was then achieved using a StatLock^®^ (Bard Access Systems, Inc., Salt Lake City, UT, USA)stabilization device. DERMABOND^®^ Mini Topical Skin Adhesive (Ethicon, Inc., Raritan, NJ, USA) was applied to ensure stable closure of the small incision site, and a BIOPATCH™ Protective Disk with CHG (Ethicon, Inc., Somerville, NJ, USA) with chlorhexidine, known for its broad-spectrum antimicrobial action, in addition to its absorptive capacity. The insertion site was finally covered with a transparent Tegaderm™ (3M Company, St. Paul, MN, USA). In cases where catheter insertion was not feasible on the first attempt, a microintroducer kit was used to facilitate the successful cannulation.

### 2.3. Modifications of Insertion Technique

**Figure 6:**
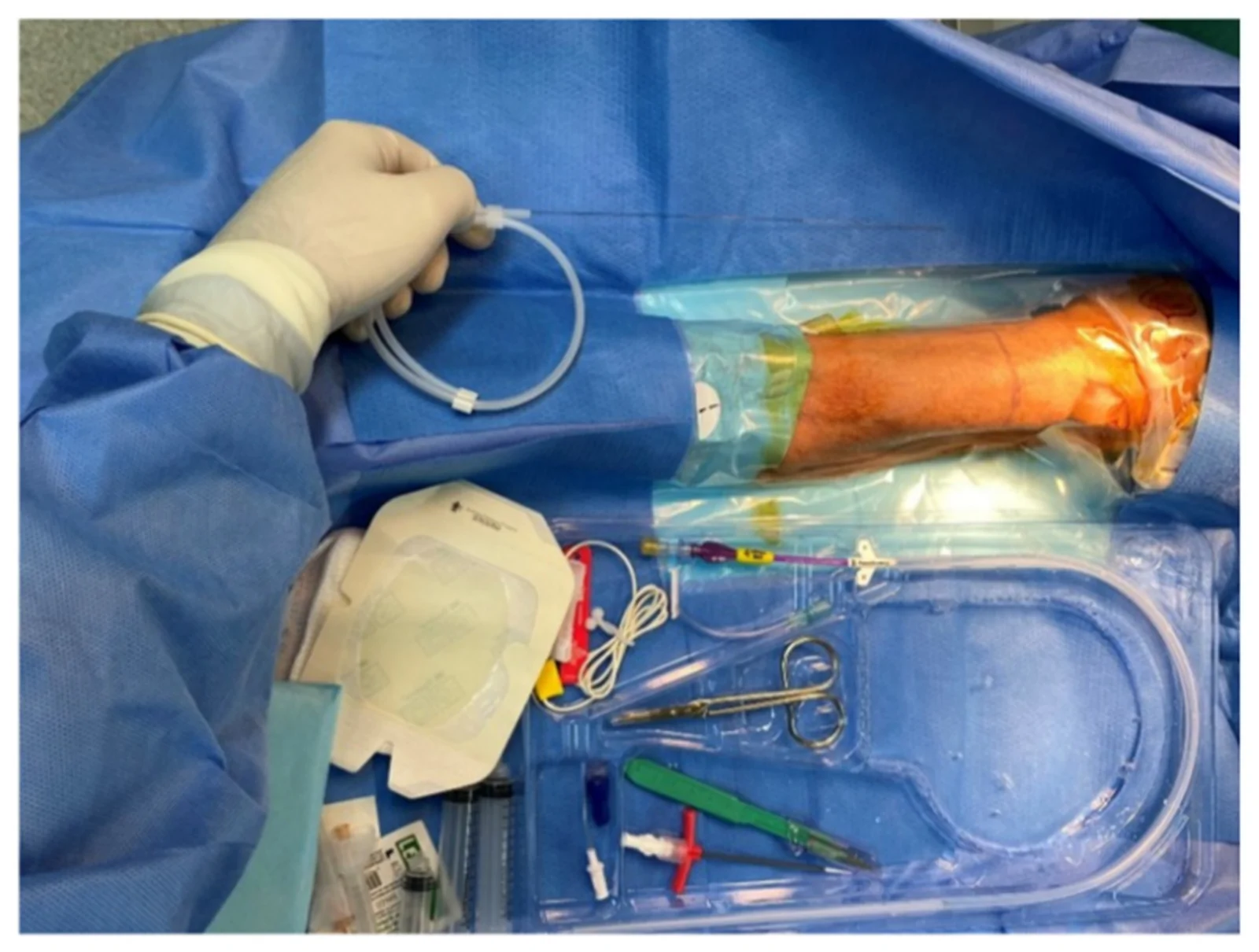
Guidewire pre-positioned for immediate insertion.

**Figure 7:**
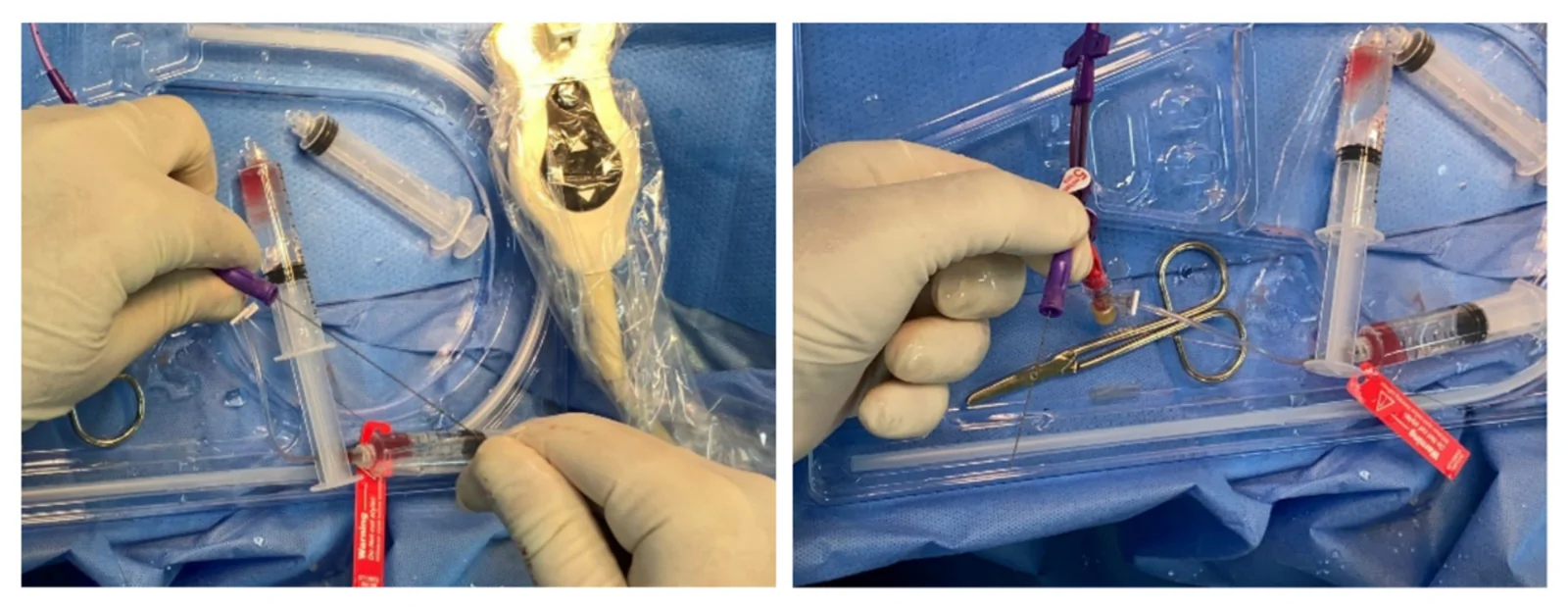
Utilization of the stiff guidewire end to facilitate catheter advancement in difficult cases.

**Figure 8:**
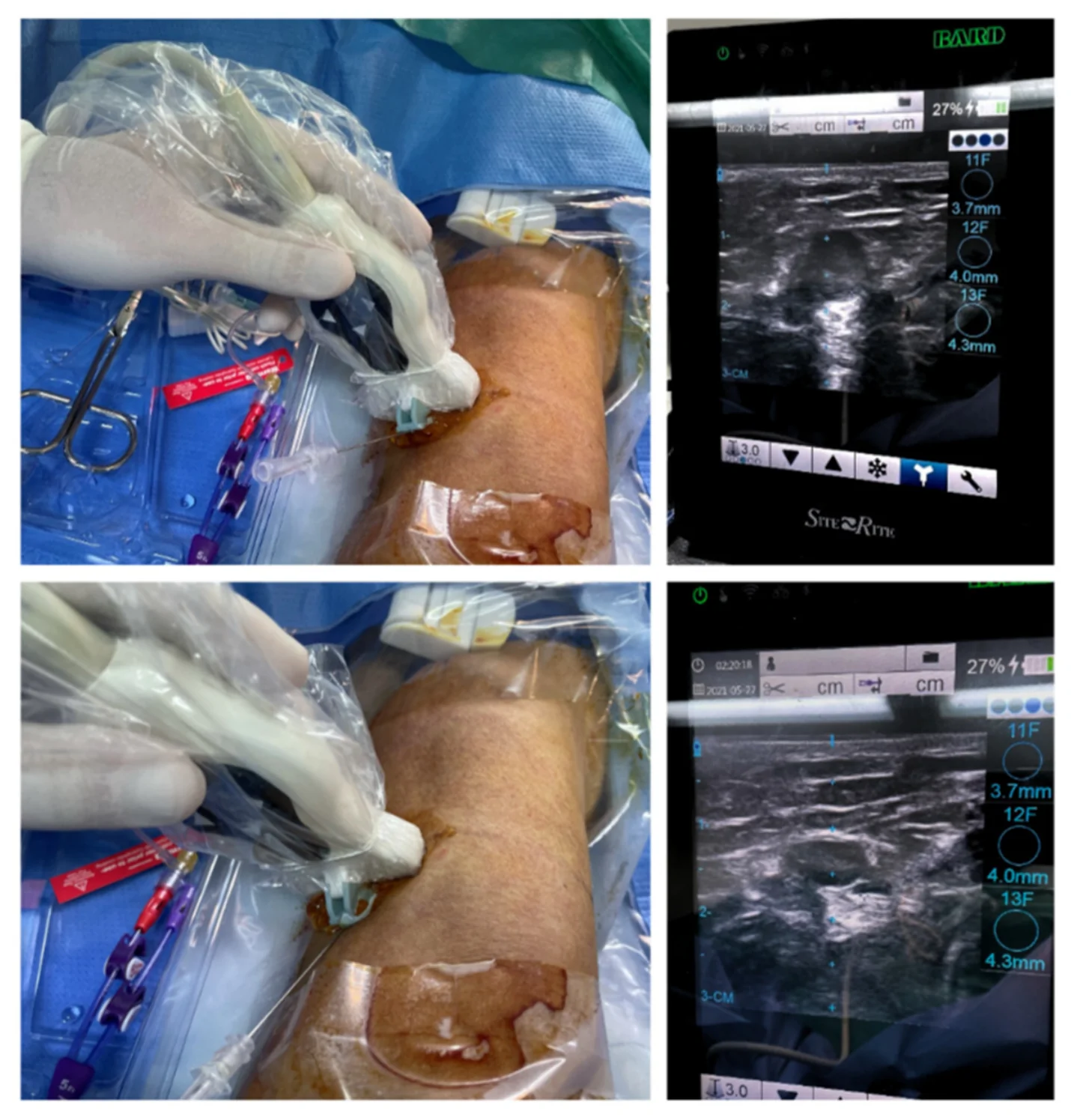
Tilting of the ultrasound transducer to facilitate cannulation of superficial veins.

Drawing on our extensive experience in establishing numerous venous access lines, we present specific procedural variations that facilitated successful outcomes in challenging cases. Optimization of guidewire: Prior to venipuncture, the guidewire was pre-positioned to provide an immediate insertion length of approximately 15 cm (Figure 6). This enabled us to insert it in a single motion, immediately after accessing the vessel, thereby avoiding unnecessary manipulations that could risk the established vascular access. Utilization of the stiff guidewire end: In patients receiving double lumen PICCs (exclusively), we employed a specific tactic when difficulties arose during the full advancement of the catheter. Once the PICC line had been advanced a few centimeters, the guidewire was withdrawn and re-inserted through the alternative lumen using its stiff end (Figure 7). This maneuver effectively stiffened the catheter, facilitating its navigation and advancement through the venous pathway. Tilting of the ultrasound: Typically, the ultrasound transducer is held perpendicular to the patient’s limb during venipuncture. However, in cases where the target vein is located very superficially, the probe is intentionally tilted. This maneuver is crucial to optimize the angle of approach and ensure successful catheterization (Figure 8).

### 2.4. Post-Procedural Management and Follow-Up

The procedure followed standard practices for PICC catheter placement, and each patient’s primary caregiver was adequately trained on proper techniques for catheter care by a specialized nurse or representative from the supplying company. Detailed instructions were provided on connecting parenteral nutrition and performing routine line maintenance, which was performed daily. These instructions included disinfection of the working surface; use of personal protective measures (including a surgical mask and hair cover); application of a sterile field on the working surface, with aseptic placement of all required materials; use of chlorhexidine solution applied with sterile gauze; thorough hand hygiene using an antiseptic solution; and donning of sterile gloves. Maintenance included flushing the catheter using a 20 mL syringe with normal saline according to the push–pause flush technique, to prevent occlusion, along with the use of a chlorhexidine wipe to clean the external portion of the line. All aspects of catheter management and maintenance were carried out at home by a specialized nurse once a week, including the change in the securement device, protective disk, and transparent film dressing.

Postoperative follow-up and monitoring for complications related to PICC placement included structured communication with patients and caregivers at predefined time points (15, 30, and 90 days), with extended follow-up up to 6 months when feasible. This structured communication protocol was implemented to enhance data consistency and early detection of complications in the home setting. Complications were assessed using a structured clinical approach, including symptom evaluation, caregiver reports, and, when feasible, bedside ultrasound assessment. In cases where hospital-based imaging was not available, diagnoses were based on clinical and ultrasound findings. Follow-up included structured interviews with caregivers and, when applicable, review of reports from the external provider responsible for the weekly catheter maintenance. The latter source provided valuable additional information mainly in cases where complications occurred (thrombosis, lumen occlusion, infection, dislodgement or accidental removal) and were communicated directly to the vascular access team.

## 3. At-Home PICC Insertion: Our Proposed Protocol

**Figure 9:**
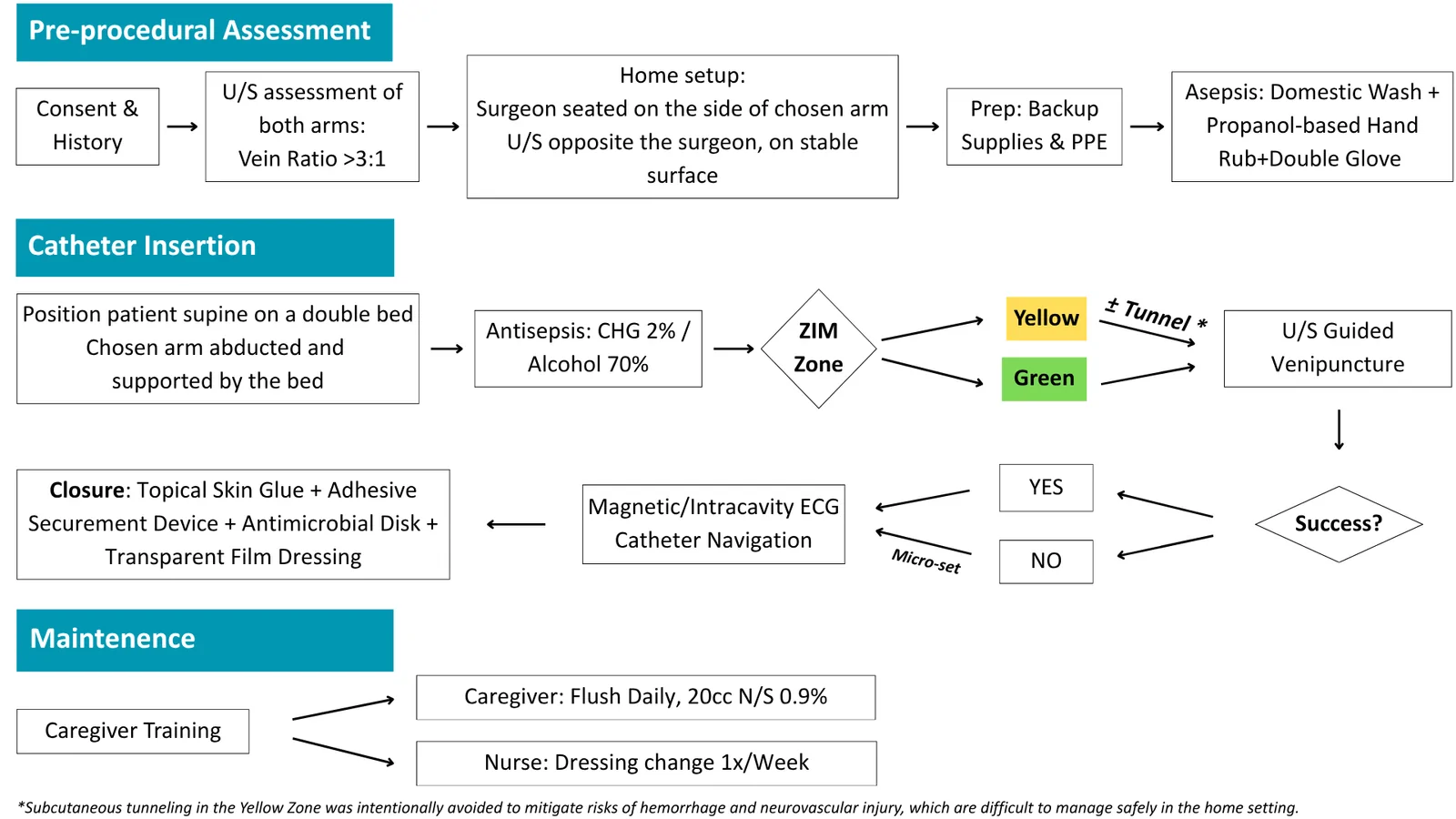
At-home PICC insertion protocol.

Developing a safe, effective, and efficient protocol demanded the evolution of our technique, based on the practical realities of the home setting. Drawing upon our cumulative experience of vascular access procedures, we created our streamlined and standardized workflow, as proposed in the following algorithm (Figure 9).

### 3.1. Feasibility and Technical Data

#### 3.1.1. Patient Selection

With regard to patient selection, it is important to first outline the criteria used to identify patients considered suitable for at-home PICC placement, thus facilitating the reproducibility of the approach. As mentioned before, life expectancy of the patient plays a vital role in the decision-making process regarding supportive care in the home setting. As a result, patients with a life expectancy of a few months or more are eligible for vascular access devices. The size of veins in cancer patients can vary greatly, thus imposing another challenge during the insertion procedure, which would be easier managed in a hospital setting. This creates the question of appropriate vascular access devices in this population group, as a small vein size in the arms could result in another method being more suited, such as a tunneled central catheter in the jugular vein. Although PICC insertion is usually classified as a low bleeding risk procedure, patients with platelet counts < 50 × 10^9^/L or INR > 2 should be excluded from at-home placement, in order to maintain a high level of safety, in accordance with international guidelines [14]. Another key aspect is the use of the intracavitary ECG guidance for the tip positioning, which also has specific limitations, mainly related to cardiac rhythm. Patients with atrial fibrillation, atrial flutter, severe atrial arrhythmias, or even pacemaker rhythm in certain cases cannot undergo this procedure at home, as an unidentifiable P-wave severely limits the accurate tip confirmation [15]. The patients included in this work represent consecutive real-world cases in whom at-home PICC placement was clinically indicated due to inability to access hospital-based care. Patients who were able to attend hospital services were managed through standard care pathways and were therefore not part of this cohort. As such, this reflects a pragmatic, indication-driven population rather than a selectively recruited sample.

To ensure the safety and feasibility of the intervention, strict patient selection criteria were applied to minimize risks associated with vascular access procedures in an uncontrolled home environment. Eligible patients were those with severe mobility and/or other logistical limitations that rendered them unable to be transferred to the hospital. All patients included in this pilot program were referred directly to the lead surgeon of our team, following a specific clinical indication for PICC placement as determined by their treating physician. This highlights a critical barrier of the current standard of care. This referral-based inclusion reflects routine clinical practice rather than predefined research selection criteria.

Exclusion criteria were implemented to safeguard patient welfare. Four individuals who had the physical ability to attend the hospital’s outpatient vascular access clinic were excluded. Patients with an estimated life expectancy of less than one month were also excluded, as the procedure was deemed disproportionate in the context of end-of-life care. Finally, financial feasibility represented an unexpected limiting factor: six patients who met clinical criteria declined participation solely due to procedural cost, highlighting a significant barrier in the current healthcare framework.

A total of 26 patients were included in this pilot initiative. Demographic characteristics are presented in Table 1. Most patients were male (57.6%), with a mean age of 74.78 years. The majority were referred for long-term parenteral nutrition, while others required reliable venous access for prolonged antimicrobial or supportive therapy.

#### 3.1.2. Technical Outcomes

The following findings reflect our real-world clinical experience with at-home PICC insertion and support the feasibility and safety considerations discussed in this perspective (Table 2). Successful catheterization of the target vein, defined as placement within the green or yellow zone according to the ZIM, was achieved in 100% of cases. A total of 28 PICCs were inserted in 26 patients. While the majority of the cohort required only a single intervention, two patients required re-insertion. One catheter was replaced following accidental removal, and a second was replaced after removal for infection, after the patient completed the therapeutic course. In both instances, the decision to re-insert was driven by the patients’ estimated life expectancy and the critical necessity for uninterrupted nutritional and therapeutic support.

Two patients required more than one insertion attempt. Notably, these difficult cases were managed without switching to the contralateral upper limb. Instead, a reassessment and repeat mapping of the available veins in the same arm was conducted to identify a vessel of adequate caliber for the intended catheter size. In two complex cases, micro-insertion sets were utilized to facilitate venous access, whereas standard PICC kits were employed for the remaining procedures.

Regarding anatomical distribution, the right arm was selected in 24 cases (85.7%), while the left arm was selected in four cases (14.3%). The basilic vein was selected in 21 patients (75%), followed by the branchial vein in seven patients (25%). Overall, 23 single-lumen and five double-lumen PICCs were inserted.

## 4. Safety and Complications

### 4.1. Infections

Four episodes of systemic infectious signs were recorded (14.29%) and an incidence rate of 1.44 events per 1000 catheter days. These manifested as fever, leukocytosis and/or elevated inflammatory markers (CRP, PCT). All events were observed later in the catheter’s lifespan, specifically on days 17, 28, 36 and 44, respectively, when a direct causal link to the insertion procedure was considered highly unlikely. Patients presenting with such symptoms were instructed by the surgeon to seek immediate evaluation, which included laboratory testing, and administration of parenteral nutrition was paused. In all cases, bacteremia was confirmed through blood cultures obtained from the PICC, and hospital admission for intravenous antimicrobial therapy was recommended. However, all four patients opted for at-home treatment with oral or ambulatory antibiotics. In all cases, the catheter was removed on days 20, 32, 41 and 47, respectively, to control the infection, and all were sent for further investigation with cultures. Following the completion of antibiotic therapy and full clinical resolution of the infection, a new PICC was successfully re-inserted in one patient to allow for the resumption of essential supportive care.

### 4.2. Thrombosis

Regarding thrombotic events, two out of 28 catheters (7.14%), corresponding to a rate of 0.72 events per 1000 catheter days, presented with localized upper-limb edema on days 42 and 55, raising suspicion for catheter-associated thrombosis. Although both patients declined hospital-based confirmation using triplex ultrasonography, thrombus formation was clinically suspected and supported by bedside ultrasound findings, using bedside B-mode compression ultrasound to assess vein compressibility. In both cases, the thrombosis was not severe enough to necessitate discontinuation of catheter use, and both PICCs were salvaged using therapeutic anticoagulation without the need for removal. Formal imaging confirmation using hospital-based modalities was not performed in all cases, primarily due to logistical constraints and patient preference.

### 4.3. Mechanical Complications

Four cases of lumen occlusion (14.29%), corresponding to 1.44 events per 1000 catheter days, were documented on days 33, 52, 59, and 67, which were initially managed using the POP (negative pressure) method [16]. This technique is used to clear occluded catheters by utilizing a three-way stopcock and a prefilled 10 mL syringe with sterile saline. The syringe is connected to one port of the stopcock, while the PICC lumen is connected to the second port. With the stopcock initially closed to the patient, the surgeon draws back on the syringe to create negative pressure. The stopcock is then rotated to open the communication between the syringe and the catheter, allowing the slight vacuum to transmit through the lumen. Next, the stopcock is turned to temporarily close the catheter and opened again toward the syringe, permitting a small-volume, low-pressure flush to be delivered. The sequence is repeated several times, producing controlled pulsatile shifts in pressure that help disengage soft deposits from the catheter tip. Although partial restoration of flow was achieved, two out of four catheters ultimately became non-functional within a few days and were removed on days 38 and 71. All complications are summarized in Table 3.

### 4.4. Dwell Time

Long-term follow-up was rather challenging in this particular cohort of patients, as much of the information was gathered from patients’ caregivers whenever contact was feasible. Despite this, we accrued a total of 2779 catheter days with a median duration of 120 days. Based on available data, catheter functionality extended up to 120 days in 19 catheters. Two deaths were recorded during the follow-up period on days 42 and 61, both unrelated to the vascular access device.

## 5. Clinical Implications and Systemic Barriers

### 5.1. Feasibility

From a perspective standpoint, the following observations should be interpreted as practice-based insights rather than definitive comparative evidence. Given the limited sample size, these findings should be interpreted with caution and are not intended to support generalized conclusions. The purpose of this work is not to establish comparative superiority over hospital-based care, but to demonstrate that at-home PICC insertion represents a feasible, effective, and safe alternative for patients with limited access to hospital services. Accordingly, feasibility is interpreted in a procedural and technical context, rather than as a comparative clinical outcome. Our experience represents one of the first documented efforts to describe the feasibility of performing PICC placement directly in the home setting. The findings of our experience align with the scarce existing literature, most notably the pilot evaluation by Keller et al. [9], confirming that the home environment does not preclude technical success when the procedure is undertaken by an experienced healthcare professional following standardized insertion protocols. This work reflects a pragmatic clinical approach, focusing on patients in whom standard hospital-based access was not feasible, rather than a comparative evaluation across different care settings. These observations should be interpreted as exploratory and hypothesis-generating, rather than definitive evidence. In several cases, the intervention enabled continuation of essential nutritional and supportive therapy in patients who would otherwise have limited access to hospital-based care, which may have contributed to prolongation of their clinical course. However, survival was not a predefined outcome and was not systematically evaluated.

### 5.2. Equipment Selection: Adapting to the Home Environment

The high technical success rate observed in this experience can be largely attributed to the strict adherence to hospital-grade protocols and to thoughtful selection of equipment tailored to support procedural autonomy and patient safety outside the clinical environment. In order to facilitate the autonomy of the surgeon and the safety of the procedure, we employed a series of technological choices.

Use of the Sherlock 3CG^®^ Tip Confirmation System played a crucial role in ensuring accurate catheter tip placement without fluoroscopy. This system combines magnetic tracking with intracavitary ECG guidance, enabling real-time visualization of the catheter tip’s trajectory and precise localization at the cavoatrial junction. The characteristic rise in P-wave amplitude provides an immediate, reliable intra-procedural indicator of correct tip positioning, thereby reducing the need for post-procedural chest radiographs. This not only streamlines the procedure but also eliminates radiation exposure and enhances overall patient safety. Literature supports the high accuracy and feasibility of 3CG systems, even in resource-limited environments, with reported tip-placement success rates comparable to fluoroscopy-guided insertions [17,18]. In our cohort, Sherlock 3CG^®^ facilitated confident, single-attempt placements in the vast majority of patients, reinforcing its value as a practical, cost-effective, and safe tool for bedside and community-based vascular access procedures [19].

The selection of the PowerGroshong^®^ PICC catheter played a pivotal role in the feasibility and safety of at-home vascular access in our patient population. Its integrated three-way valve mechanism, which remains closed under neutral pressure and opens only during infusion or aspiration, significantly reduces the risk of blood reflux and air embolism. Those are key concerns in non-hospital environments. Furthermore, its maintenance requirements are notably minimal, requiring only weekly saline flushes without the need for heparin, thereby reducing the risk of heparin-related complications and simplifying the responsibilities of the caregivers. Constructed from biocompatible silicone, the device is well-suited for long-term indwelling, a crucial feature in palliative and oncology settings [20]. Additionally, all components are latex-free, minimizing the risk of hypersensitivity reactions. Importantly, the catheter is rated for power injection, allowing its use in contrast-enhanced imaging without necessitating additional venous access, if need presents. This feature is also present in the double-lumen PowerPICC™ Catheter, which offers the advantage of simultaneous administration of incompatible medications or therapies through separate channels, reducing the need for multiple venous accesses [21].

### 5.3. Procedural Considerations

Several procedural decisions regarding selection of the number of lumens, the vein, or even the arm of choice were carefully considered and likely contributed to the high rate of procedural success. More specifically, selection of the right arm provides an easier venous pathway, as the right brachiocephalic vein offers a more direct route to the superior vena cava compared to the left, facilitating easier catheter advancement and reducing the risk of malposition [22]. The basilic vein was the vessel of choice in most patients, due to its sufficient diameter to accommodate PICC lines, ensuring adequate blood flow around the catheter and reducing the risk of thrombosis, and suitability for ultrasound-guided access, which enhances placement accuracy and safety. Finally, in regard to the number of lumens, the lowest possible number is always the preferred option: single-lumen catheters were used whenever clinically acceptable, with double-lumen devices reserved only for patients requiring concurrent administration of incompatible medications or therapies. This strategy aligns with evidence indicating lower infection and thrombosis risk with fewer lumens [23].

### 5.4. The Infection Paradox: Insertion vs Maintenance

While our insertion outcomes were excellent, complication rates in our experience revealed a significant difference compared to hospital-based reports [24]. In Kang et al.’s prospective cohort of 477 cancer patients [25], the incidence of central line-associated bloodstream infection (CLABSI) was 1.3%. Similarly, Grau et al. [26] reported a 6.3% bloodstream infection rate in a prospective cohort of 192 catheters. In contrast, our cohort experienced a higher rate of infection (14.29%).

However, a deeper analysis of the data offers a critical perspective, as several factors likely contributed to these elevated complication rates. Firstly, our population consisted largely of a vulnerable group of frail oncology patients, in whom malnutrition, immobility, and comorbidities predispose to catheter-related complications. Unlike hospital-based series, where PICCs are maintained by trained professionals, catheter care in our initiative relied primarily on patients’ caregivers and representatives of the supplying company, with professional nursing input limited to weekly visits. The lack of standardized, professional maintenance and variability in care quality may have contributed to the higher observed infection rate. Notably, all bacteremic episodes occurred beyond 15 days after insertion, which, based on their delayed onset (>15 days), may suggest a non-procedural origin. In vascular access practice, early complications (typically within the first 5–7 days) are more often associated with insertion technique, whereas later events are generally related to catheter use and maintenance. Importantly, similar complication patterns were reported in patients with hospital-inserted PICCs who are subsequently managed in non-hospital settings, suggesting that complication risk is more closely related to maintenance conditions than to the insertion environment itself. However, this interpretation is based on temporal association and does not establish a definitive causal relationship. However, in healthcare systems where such structured services by dedicated vascular access teams are not available, pragmatic strategies must also be considered in order to safely manage the current reality, in which catheter care may often rely on patients’ relatives or informal caregivers. Given the descriptive nature of the data, any distinction between insertion-related and maintenance-related complications should be interpreted as hypothesis-generating rather than definitive and within the context of real-world care conditions, including patient frailty and non-standardized catheter maintenance.

Similarly, the incidence of clinically suspected thrombotic events in our high-risk oncology cohort (7.14%) exceeds the pooled rates reported in recent meta-analyses, where PICC-associated thrombosis risk is generally reported between 2% and 5% [27]. However, it must be noted that our estimate reflects clinical assessment rather than radiologically confirmed diagnoses. This observation nonetheless aligns with the well-recognized thrombogenic potential of PICCs compared to other central devices, particularly in high-risk populations [28].

Therefore, the concept of safety in this setting should be interpreted in relation to procedural feasibility and real-world applicability, rather than as equivalence to hospital-based outcomes.

### 5.5. The Necessity of Professional Certification

At-home PICC insertion is an advanced practice that requires a high level of procedural expertise and rigorous safety protocols. In this context, the specialized surgeon who performed the insertions has attended multiple national and international training courses in venous access, including courses organized by Global Vascular Access Network/World Congress on Vascular Access (GloVANet/WoCoVA). At the initiation of this pioneering effort, the surgeon’s experience included approximately 200 PICC insertions, whereas by the time the final procedure in this series was performed, this number had exceeded 2500 PICCs.

For this model to be safely scaled at a national scale, formal credentialing and clear institutional governance frameworks need to be established [29]. This necessity raises an important ethical and logistical question: who assumes responsibility for the procedure when it is performed outside the hospital, within the patient’s home? It is imperative to consider ensuring professional accountability, safe working conditions, and clear legal protections before widespread implementation of home-based vascular access programs.

Beyond technical competence, the performance of invasive procedures in the home setting raises important ethical and governance considerations. These include ensuring patient safety in a non-controlled environment, maintaining clear professional accountability, and obtaining fully informed consent under conditions that differ from hospital-based care. Furthermore, the absence of structured institutional oversight highlights the need for formal governance frameworks, including certification standards, defined clinical responsibilities, and medico-legal protection for healthcare providers. Addressing these elements is essential for the safe and ethical expansion of home-based vascular access services.

### 5.6. Inequity in Access: The Financial Burden of Home Care

At this point, it is important to address some considerations regarding the economic aspects of at-home PICC placement. Unfortunately, while other consumables such as stoma bags and ostomy products are reimbursed by public insurance, national insurance schemes do not currently reimburse patients for PICC insertion performed in the home setting. With a total cost approaching approximately €1000, including materials and professional services, this intervention remains financially inaccessible for a substantial proportion of patients who could benefit from such an intervention. In the absence of public reimbursement, cost becomes a prohibitive barrier, effectively creating inequity in access to advanced vascular care. In contrast, PICC insertion performed in the hospital setting is typically covered by public healthcare systems and is provided without direct cost to the patient, highlighting a significant disparity in access between hospital-based and home-based care. An alternative strategy worth consideration is the use of mobile medical units equipped for vascular access procedures, which may provide a more controlled environment compared to in-home placement and could potentially improve both procedural safety and cost-effectiveness. However, further evaluation is required to determine whether such benefits can be consistently achieved in different healthcare settings. It is essential to establish a structured and legally supported framework that allows for the expansion of vascular access services beyond hospital settings while ensuring optimal patient care and procedural safety.

It should be highlighted that no formal economic analysis was performed, and the cost estimates presented are descriptive; therefore, no conclusions regarding cost-effectiveness can be drawn from the available data.

## 6. Limitations

We acknowledge several limitations of this work. First, the data presented are derived from real-world clinical practice and were reviewed retrospectively, which may limit completeness and introduce potential bias. In addition, the relatively small patient cohort, drawn from a single regional program, restricts the generalizability of these observations. In particular, the sample size is insufficient to draw definitive conclusions regarding complication rates or safety outcomes. Importantly, these data were not initially collected within the framework of a predefined research protocol but rather accumulated during routine clinical care and later analyzed descriptively to support this perspective. As such, the findings should be interpreted as practice-based insights rather than definitive comparative evidence. Finally, the limited number of procedures reflects real-world logistical and financial constraints. In several cases, the operating surgeon was required to travel considerable distances (up to 150 km) to reach patients across Northern Greece, which inherently restricted procedural volume.

Second, follow-up data were primarily accumulated through patient caregivers, and, to a lesser extent, reports from supplying companies, which may have introduced reporting bias. Importantly, the findings apply to a specific subgroup of patients with significant mobility or logistical limitations requiring home-based care and therefore may not be generalizable to the broader oncology population. Additionally, certain complications, particularly thrombotic events, were not systematically confirmed using standard hospital-based imaging, which may limit diagnostic accuracy and introduce potential misclassification. Furthermore, although temporal patterns were used to inform clinical interpretation, the available data do not allow for a definitive distinction between insertion-related and maintenance-related complications. Patient-centered outcomes, including quality of life, satisfaction, and survival, were not formally assessed using standardized methodologies and therefore cannot be quantitatively evaluated in this work.

Additionally, the data were derived from multiple real-world sources, including routine clinical practice, caregiver reports, and external providers, which may introduce variability in data completeness, as well as potential reporting and measurement inconsistencies, particularly in the assessment of complications. Last but not least, the implementation of a structured communication protocol with follow-up extending up to 6 months aimed to improve data consistency and mitigate these limitations. “Additionally, the relatively high cost of the procedure limits accessibility and precludes conclusions regarding cost-effectiveness.”

Finally, the non-standardized nature of catheter maintenance, performed by caregivers with varying levels of training and experience, makes it challenging to definitively record complications attributed to the insertion technique, rather than daily catheter care. During catheter placement, all necessary precautions recommended by international infection-prevention guidelines were followed in order to minimize the risk of catheter-related infections. However, the operating surgeon was not formally responsible for the education of patients’ relatives or caregivers regarding catheter care. Nevertheless, these limitations accurately reflect the real-world challenges inherent to delivering advanced medical procedures in the Greek home-care setting.

## 7. Conclusions and Future Directions

At-home PICC insertion in cancer patients appears to be a feasible and clinically applicable approach when performed by experienced professionals under strict procedural standards. The observations presented in this perspective, supported by real-world data, suggest that this model may contribute to improved continuity of care and patient-centered service delivery in selected populations. It should be highlighted that these observations are descriptive and should not be interpreted as evidence of superiority over standard hospital-based care. These findings should therefore be considered preliminary and hypothesis-generating. Furthermore, no comparison with hospital-based PICC placement was performed or intended within the scope of this work.

However, these findings should be interpreted within the context of a perspective based on clinical experience rather than definitive comparative evidence. Further studies, ideally involving larger cohorts and standardized methodologies, are required to better define safety, effectiveness, and broader applicability.

## Figures and Tables

**Table 1:** Demographic characteristics of 26 patients with PICCs placed at home.

Variable	Total (Percentage of *n* = 26)
Age (mean, median, IQR)	74.78, 75, (67–82)
Sex	
Male	15 (57.6%)
Female	11 (42.3%)
Race/Ethnicity: White non-Hispanic	26 (100%)
Geographic Region: Northern Greece	26 (100%)

**Table 2:** Outcomes of patients with 28 PICCs placed at home.

Variable	Total (Percentage of *n* = 28)
Catheterization within green/yellow zone (according to ZIM)	28 (100%)
Green Zone	26 (92.9%)
Yellow Zone	2 (7.1%)
Catheterization on the first attempt	26 (92.9%)
Catheterization of the right arm	24 (85.7%)
Catheterization of the left arm	4 (14.3%)
Type of catheter used	
Single lumen	23 (82.1%)
Double lumen	5 (17.9%)
Vein of choice	
Basilic vein	21 (75%)
Branchial vein	7 (25%)

**Table 3:** Complications of patients with 28 PICCs placed at home.

Variable	Total (Percentage of *n* = 28)
Admissions within 30 days of PICC placement	0 (0.0%)
Catheter inadvertently removed	1 (3.57%)
Catheter leakage	0 (0.0%)
Possible venous thromboembolism	2 (7.14%)
Catheter occlusion	4 (14.29%)
Bloodstream infection	4 (14.29%)
Any catheter complication	10 (35.7%)

## Data Availability

The data supporting the findings of this work are available from the corresponding author upon reasonable request, due to privacy and ethical restrictions.

## Abbreviations

The following abbreviations are used in this manuscript:

PICC	Peripherally inserted central catheter
ECG	Electrocardiogram
ZIM	Zone Insertion Method
CVAD	Central venous access device
CRP	C-reactive protein
PCT	Procalcitonin
UEDVT	Upper extremity deep venous thrombosis
CLABSI	Central line-associated bloodstream infection
ESPEN	European Society for Clinical Nutrition and Metabolism
PICO	Population, Intervention, Comparison, Outcome
SIP	Safe insertion of PICCs
TCS	Tip Confirmation System
POP	PICC Occlusion Protocol
GloVANet	Global Vascular Access Network
WoCoVA	World Congress on Vascular Access
